# Diversity of Human Enterovirus Co-Circulations in Five Kindergartens in Bangkok between July 2019 and January 2020

**DOI:** 10.3390/v15061397

**Published:** 2023-06-20

**Authors:** Pichamon Sittikul, Elizabeth M. Batty, Prasert Yodsawat, Jiratchaya Nuanpirom, Nathamon Kosoltanapiwat, Unitsa Sangket, Supawat Chatchen, Nicholas P. J. Day, Janjira Thaipadungpanit

**Affiliations:** 1 Department of Tropical Pediatrics, Faculty of Tropical Medicine, Mahidol University, Bangkok 10400, Thailand; pichamon.sit@mahidol.ac.th (P.S.); supawat.cht@mahidol.ac.th (S.C.); 2 Mahidol-Oxford Tropical Medicine Research Unit, Faculty of Tropical Medicine, Mahidol University, Bangkok 10400, Thailand; elizabeth.b@tropmedres.ac (E.M.B.); prasert.y@outlook.com (P.Y.); nickd@tropmedres.ac (N.P.J.D.); 3 Centre for Tropical Medicine and Global Health, Nuffield Department of Clinical Medicine, University of Oxford, Oxford OX3 7LG, UK; 4 Division of Biological Science, Faculty of Science, Prince of Songkla University, Songkhla 90110, Thailand; jirath.nuan@gmail.com (J.N.); unitsa.s@psu.ac.th (U.S.); 5 Department of Microbiology and Immunology, Faculty of Tropical Medicine, Mahidol University, Bangkok 10400, Thailand; nathamon.kos@mahidol.ac.th; 6 Center for Genomics and Bioinformatics Research, Faculty of Science Prince of Songkla University, Songkhla 90110, Thailand; 7 Department of Clinical Tropical Medicine, Faculty of Tropical Medicine, Mahidol University, Bangkok 10400, Thailand

**Keywords:** hand–foot–mouth diseases, pediatrics, infectious disease, Enterovirus A71, whole genome sequencing, SISPA, Oxford Nanopore Technology

## Abstract

Human enterovirus causes various clinical manifestations in the form of rashes, febrile illness, flu-like illness, uveitis, hand–foot–mouth disease (HFMD), herpangina, meningitis, and encephalitis. Enterovirus A71 and coxsackievirus are significant causes of epidemic HFMD worldwide, especially in children aged from birth to five years old. The enterovirus genotype variants causing HFMD epidemics have been reported increasingly worldwide in the last decade. We aim to use simple and robust molecular tools to investigate human enteroviruses circulating among kindergarten students at genotype and subgenotype levels. With the partial 5′-UTR sequencing analysis as a low-resolution preliminary grouping tool, ten enterovirus A71 (EV-A71) and coxsackievirus clusters were identified among 18 symptomatic cases and 14 asymptomatic cases in five kindergartens in Bangkok, Thailand, between July 2019 and January 2020. Two occurrences of a single clone causing an infection cluster were identified (EV-A71 C1-like subgenotype and coxsackievirus A6). Random amplification-based sequencing using MinION (Oxford Nanopore Technology) helped identify viral transmission between two closely related clones. Diverse genotypes co-circulating among children in kindergartens are reservoirs for new genotype variants emerging, which might be more virulent or better at immune escape. Surveillance of highly contagious enterovirus in communities is essential for disease notifications and controls.

## 1. Introduction

Hand, foot, and mouth disease (HFMD) and herpangina are common pediatric illnesses affecting children from birth to five years old. The symptoms range from mild to severe illness, with encephalitis leading to death. Common symptoms are acute fever, sore throats, malaise, and poor feeding. HFMD presents with vesicular rashes or blisters on palms, soles, buttocks, and oral mucosa, while herpangina presents with small ulcerate or vesicular lesions in the posterior oropharynx [1,2]. The diseases are transmitted through an oral–faecal route by ingesting food or water contaminated with infected saliva, respiratory droplets, or fluid from vesicles. Infections are highly contagious in the first 14 days. Symptomatic infections manifest 3–5 days after exposure. Over 20 human enterovirus serotypes have been reported as causing HFMD and herpangina. Coxsackievirus and echovirus can cause either mild infections, such as herpangina, or severe manifestations, including sepsis, hepatitis, myocarditis, encephalitis, meningitis, and multi-organ failures, leading to death in neonates and young infants [3,4,5]. The human enterovirus belongs to the Genus *Enterovirus* of the Family *Picornaviridae* [6]. It is a single positive-stranded RNA virus with a 7.4-kilobase genome. Eleven proteins are coded under a single open reading frame containing four structural capsid proteins (VP1–VP4) and seven nonstructural proteins (2A–2C and 3A–3D) franked with 5′ and 3′ untranslated regions.

HFMD is a public health threat that has caused several large severe outbreaks across Europe, North America, and Asia. HFMD is considered an endemic disease in the Asia-Pacific, including Cambodia, China, Malaysia, Singapore, Taiwan, Thailand, and Vietnam, with an estimated annual incidence of over two million cases [7]. The Enterovirus A71 (EV–A71) and Coxsackievirus A (CVA) groups are the primary causative agents of HFMD worldwide. In Thailand, HFMD has been listed as a disease requiring notification to the Thai Ministry of Public Health (MOPH) since 2001 [8]. Each year, HFMD cases peak in the wet season (June and September). A large-scale outbreak of HFMD was first reported in 2012, affecting over 39,000 patients (62.7/100,000 person years) [9]. EV–A71, CVA6 and CVA16 were significant causative agents of HFMD between 1957 and 2017 [8,10,11]. Enterovirus infections could cause severe illness leading to death in neonates and infants. Severe neonatal sepsis cases increased from 6.2% (between 2016 and 2021) to 55% (July 2022 to April 2023) in France [12]. The new emerging echovirus E11 caused severe sepsis with hepatic impairment and multi-organ failure, associated with a fatality rate of 78%.

In the next-generation sequencing era, whole genome sequencing (WGS) has been used to study the molecular epidemiology of viral diseases, viral infectivity, transmissibility, and virulence [13,14,15]. Sequence-independent, single-primer amplification (SISPA) is a widely used technique for viral and bacterial whole genome sequencing, including influenza, chikungunya, dengue, SARS-CoV-2, and uncommon enterovirus isolates [16,17,18,19,20]. SISPA uses a random priming method allowing for viral and bacterial genome enrichment via a short process. Short-read and long-read sequencing platforms can be used with enriched genomic DNA. Oxford Nanopore Sequencing Technology is the third-generation sequencing platform. The size of the portable machine, MinION, is similar to that of a smartphone. One of the limitations is a higher error rate in the sequence data compared to short-read sequencing technologies. The use of the platform has increased because of the affordable price of flowcells, a variety of library preparation kits and the maintenance-free system design. This system is handy for use in resource-limited laboratories [21].

In 2019–2020, we investigated transmission patterns of HFMD and herpangina in kindergartens in metropolitan areas of Bangkok, Thailand. The estimated HFMD incidence from the study was 246.63/10,000 person years, which was distinctively higher than those of Thai MOPH reports due to our active surveillance [22]. While EV–A71 was the most common human enterovirus detected among symptomatic infections and asymptomatic infections, as reported before, the other predominant human enterovirus included CVA and Echoviruses (Echo) groups. Based on genetically inferred serotypes, 45% of the subsequent infections were the same serotypes as the first index case in each infection cluster. However, the study did not describe the genetic relatedness among symptomatic and asymptomatic cases, potential transmission of the virus between classes, and the in-host variants of virus RNA shedding in the follow-up specimens [22].

### Objectives

In this study, we investigated human enterovirus co-circulation in kindergarten students which caused symptomatic (HFMD and herpangina) and asymptomatic infections based on single-gene typing using data, clinical specimens, and virus isolates from the prospective cohort study of five kindergartens in metropolitan Bangkok, Thailand. Four hundred and nine bases of conserved five prime untranslated regions (5′-UTR) of the RNA virus and the complete viral genomes acquired from the GenBank database were used for the genotype evaluation. We also investigated genotype changes among viral RNA shedding in single index cases and the viral RNA shedding duration in clinical samples in HFMD and herpangina cases and whether whole genome sequencing can be helpful in increasing our ability to detect enterovirus transmission.

## 2. Materials and Methods

### 2.1. Cohort Study and Clinical Samples

The prospective cohort study investigating the prevalence of HFMD in a kindergarten-based setting in Bangkok, Thailand, between June 2019 and Jan 2020 was reported previously [22]. Briefly, throat and blister swabs from HFMD and herpangina participants, clinically diagnosed by physicians, were collected at their houses at the first visits. These students were assigned as “index cases” in classrooms at given reported times called “events”. In addition, index cases were asked for throat swabs and stool collections every week for another four weeks. Within the first three days after the index cases were reported, eight recruited classmates in the same classroom with index cases (called case contacts) self-voluntarily allowed us to collect throat swabs from them at school. For sequencing and genotyping analysis, the current study used specimens from all visits of the selected 18 index cases and 14 case contacts with confirmed virus infections. Before the virus culture, all swabs were preserved in a viral or universal transport media (UTM) (COPAN, CA, USA), while the throat swabs and stool samples were stored at −80 °C until used for genotyping. The Faculty of Tropical Medicine Ethics Committees, Mahidol University, approved the study (protocol no. TMEC 19-017). All data are confidential and anonymous.

### 2.2. Detection of Enterovirus Infections Based on Real-Time RT PCR

Throat swabs from the first visit of the selected 18 index cases and 14 case contacts had been screened for enterovirus infections using the molecular method and virus isolation as reported earlier [22]. This study screened all available clinical specimens from further follow-up visits for enterovirus infections. Briefly, RNA samples were prepared from clinical specimens, including throat swabs and stool and blister swabs using a QIAamp Viral RNA Mini Kit (Qiagen, Hilden, Germany). To confirm enterovirus infections, a one-step real-time reverse transcription polymerase chain reaction (RT-PCR) with primers and hydrolysis probes targeting the conserved region of 5′-UTR was performed [22,23]. The PCR assay modification was as follows: qScript XLT one-step rt-qpcr toughmix (Quantabio, MA, USA) was used as the master mix, and each reaction had ten pmol each of forward EQ1 (5′-ACATGGTGTGAAGAGTCTATTGAGCT-3′) and reverse primers EQ2 (5′-CCAAAGTAGTCGGTTCCGC-3′) and 2.5 pmol of probe EPmod (5′-ATTAGCCGCATTCAGGGGCCGGA-3′). A 20 µL reaction mixture contained 4 µL of RNA samples. The assay was performed using the Touch Real-Time PCR Detection System, CFX96 (Bio-Rad, CA, USA) at the Faculty of Tropical Medicine laboratory and with the following cycling conditions: 50 °C for 10 min as a reverse transcription step, 95 °C for 1 min, followed by 45 cycles of 95 °C for 5 s and 62 °C for 30 s. The intensity of 6-carboxy-fluorescein (FAM) was acquired at the end of each 62 °C step. A positive control (EV-A71 virus RNA) and negative control (nuclease-free water) were included in each screening batch. A positive PCR result is defined when the reaction shows a FAM signal above a fixed threshold of 500.

### 2.3. Genotyping Enterovirus A and B Based on 5′-UTR Sequence Analysis

The follow-up visit specimens, which were real-time RT-PCR-positive, were genotyped based on the conserved partial 5′-UTR sequences. RT-PCR and nested PCR were performed, and the amplicons were purified before sending for Sanger DNA sequencing (Macrogen, Seoul, Republic of Korea), as published previously [22]. The chromatogram results were inspected for consensus between the sequences from the reverse and forward sequencing primers using the Molecular Evolutionary Genetics Analysis (MEGA X) software [24]. Maximum likelihood trees were reconstructed from the trimmed 410-nucleotide 5′-UTR region alignments (from position 183 to 532 based on EV-A71 sub.B5 GenBank accession number: JN964686) or the trimmed 6919-nucleotide complete genome on the General Time-Reversible model to infer species and genetic relatedness using MEGA X. The number of base differences per sequence from averaging over all sequence pairs within each group was estimated. All 5′-UTR sequences from this cohort (previously reported as MZ004661 to 693, and in the current study as OQ304497 to 505) (Supplementary Data S1) were submitted to the GenBank database. The complete genome sequences of *Enterovirus A* and *Enterovirus B* acquired from GenBank data used for the analysis were shown in Supplementary Data S2.

### 2.4. Viral Whole Genome Sequencing for a Double Infection Case

Two virus isolates from throat swabs taken during the 1st visit and 4th visit of index case No 7, which belonged to two different species of genus *Enterovirus*, were investigated based on the SISPA technique of constructing the genomic amplified complementary DNA (cDNA) and the long read whole genome sequencing using MinION (Oxford Nanopore Technologies, Oxford, UK) as reported previously with modification [18,25]. Double-stranded DNA was needed to prepare nanopore sequencing libraries. The primer extension preamplification method comprising of “Round A” (RNA reverse transcription followed by second-strand DNA synthesis) and “Round B” (DNA amplification) has been used for amplifying organism genomes randomly using the following primers: sol-primer A (5′-GTTTCCCACTGGAGGATA (N_9_)-3′) and sol-primer B (5′-GTTTCCCACTGGAGGATA-3′). Briefly, the Round A protocol was performed as described earlier by Greninger [20], and Round B was modified in the following way. The 7.5 µL of the Round A products were added into a 25 µL amplification reaction containing LongAmp Taq PCR Mix (New England Biolabs, Ipswich, MA, USA) and 50pmol of sol-primer B. The assay was performed using the Touch Real-Time PCR Detection System, CFX96 (Bio-Rad, Hercules, CA, USA) at the Faculty of Tropical Medicine laboratory and with the following cycling conditions: 94 °C for 2 min followed by 25 cycles of 94 °C for 30 s, 52 °C for 45 s, 65 °C for 60 s, and a final extension at 65 °C for 5 min. The DNA products were purified using AMPure XP beads (Beckman Coulter, Brea, CA, USA) then then quantified using a Qubit dsDNA HS assay (Invitrogen, Carlsbad, MA, USA). The nanopore sequencing libraries with multiplexing samples were prepared using the 500 ng of DNA, the Ligation Sequencing Kit (SQK-LSK109) and Native Barcoding Expansion Kit (EXP-NBD104) (Oxford Nanopore Technologies, Oxford, UK) as described by the manufacturer. The final sequencing library at 50–70 fmol was an input for the long-read sequencing device (MinION Mk1c) (Oxford Nanopore Technologies, Oxford, UK) using R9.4.1 flow cells (FLO-MIN106) (Oxford Nanopore Technologies, Oxford, UK).

The direct cDNA sequencing kit (SQK-DCS 109; Oxford Nanopore Technologies, Oxford, UK) was used for sequencing RNA full-length transcripts as described by the manufacturer. Briefly, 100 ng of poly(A+) RNA was reverse-transcribed and strand-switched using Maxima H Minus Reverse Transcriptase (Thermo Scientific, Waltham, MA, USA) and primers supplied with the sequencing kit. The RNA templates were then degraded using an RNase Cocktail Enzyme Mix (Invitrogen, Carlsbad, MA, USA), and the second strand synthesis was performed using a LongAmp Taq PCR Mix (New England Biolabs, Ipswich, MA, USA). Then, the nanopore sequencing libraries with multiplexing samples were performed using the Native barcoding expansion kit (Oxford Nanopore Technologies, Oxford, UK). As described above, 60 ng of the cDNA library was used for long-read sequencing.

### 2.5. Nanopore Sequencing Data Analysis

Bioconda software [26] was used to manage the software installation and environment. Nanopore sequencing data were base-called and demultiplexed using Guppy software (v.6.3.7). Sequencing adapters were trimmed using Porechop software (v.0.2.4) (https://github.com/rrwick/Porechop accessed on 1 June 2022) and reads with middle adapters were also discarded (--discard_middle). For the samples with SISPA amplification, the SISPA primer sequences were removed using Cutadapt software (v.4.1) [27]. Reads with an average quality score of less than eight and a length of less than 200 bp were filtered using NanoFilt software (v.2.8.0) [28]. Human genome contamination was removed by mapping reads against human genome GRCh38.p13 using Minimap2 software (v.2.24) [29,30] and then selecting unmapped reads from the alignment file using SAMtools software (v.1.15.1) [31]. To assess the completeness of the enterovirus genome, the remaining reads were mapped to the enterovirus A71 genome (GenBank: U22521.1) for Visit 1 index case No. 3 and Visit 1 index case No. 7; and to the enterovirus B genome (RefSeq: NC_001472.1) for Visit 1 contact case No. 85 and Visit 4 index case No. 7 using Minimap2 software. Genome coverage and read depth were calculated using SAMtools. For the clinical samples, the remaining reads were mapped to the genomes of human enteroviruses retrieved from the NCBI virus database on 6 October 2022. Briefly, we searched for the taxonomy ID 12059 and filtered for complete human enterovirus genomes. The genome coverage and read depth were calculated based on the alignment result that achieved the highest genome coverage. Variant calling was performed using BCFtools (v.1.16) software [32], and variants were filtered using bedtools software [33] to remove those with a read depth below 15 reads. Consensus sequences were generated using BCFtools [32] and SeqKit (v.2.3.1) [34] software.

## 3. Results

### 3.1. Genotyping of the Enterovirus Infections Based on Partial Sequences of the Five Prime Untranslated Region

**Figure 1:**
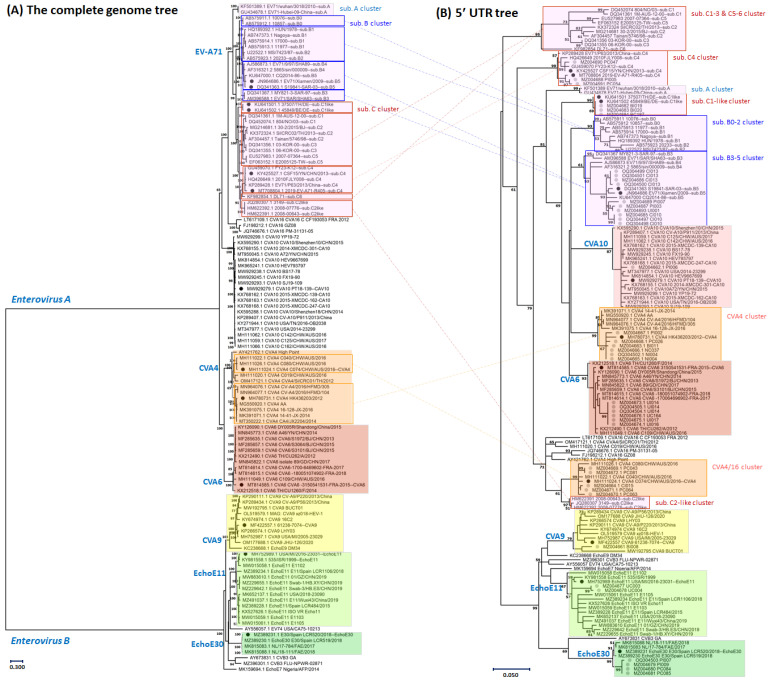
The phylogenetic tree on panel (**A**) was reconstructed based on 6,919 bases of 116 reference complete enterovirus genomes, while the tree on panel (**B**) was reconstructed based on 410 bases of the 5′-UTR region from 41 clinical specimens (18 index cases and 14 case contacts) in this cohort study and reference sequences from trimmed complete genomes. The maximum likelihood method and General Time-Reversible model were used to estimate genetic relatedness. The percentage of trees (over 50%) in which the associated taxa clustered together is shown above the branches. The scale bars show substitutions per site: (**A**) 30% and (**B**) 5%. The enterovirus genotype clusters were compared between two trees. The EV-A71 genotypes B and C were separated into two and four clusters on the 5′-UTR tree, respectively. The CVA4 genotypes were divided into two clusters on the 5′-UTR tree: CVA4 and CVA4/16.

The EV-A71 sub-genotype clusters inferred from the 5′-UTR maximum likelihood tree were the same as those inferred from the complete genome tree. Both trees used 37 reference sequences (including previously reported [35]) to sub-genotype the EV-A71, as shown in Figure 1. However, the EV-A71 genotypes (A, B, and C) were not grouped into a single cluster based on the partial 5′-UTR region when compared to the complete genome tree. The C1-like and C2-like sub-genotype infections were more distantly related to other sequences from genotype C than non-genotype C sequences, which was confirmed by both trees. Similar to EV-A71, each Coxsackievirus group of *Enterovirus A* (the 48 reference genome sequences of CVA4, CVA6, CVA10, and CVA16) was clustered into its clade in both trees, except that CVA4 resolved into two groups in the 5′-UTR tree. One group clustering with Australian isolates (MH111024) was more related to CVA16 than the other CVA4 group clustering with the Hong Kong isolate (MH780731), so-called CVA4/16. Lastly, the *Enterovirus B* branch comprised 31 genome sequences and demonstrated that CVA9, Echovirus E11 (EchoE11), and Echovirus E30 (EchoE30) were grouped into clusters on either the 5′-UTR or the complete genome trees (Figure 1). These results indicate that the partial 5′-UTR region has sufficient resolution to accurately infer human enterovirus species and subspecies with proper reference sequences.

**Figure 2:**
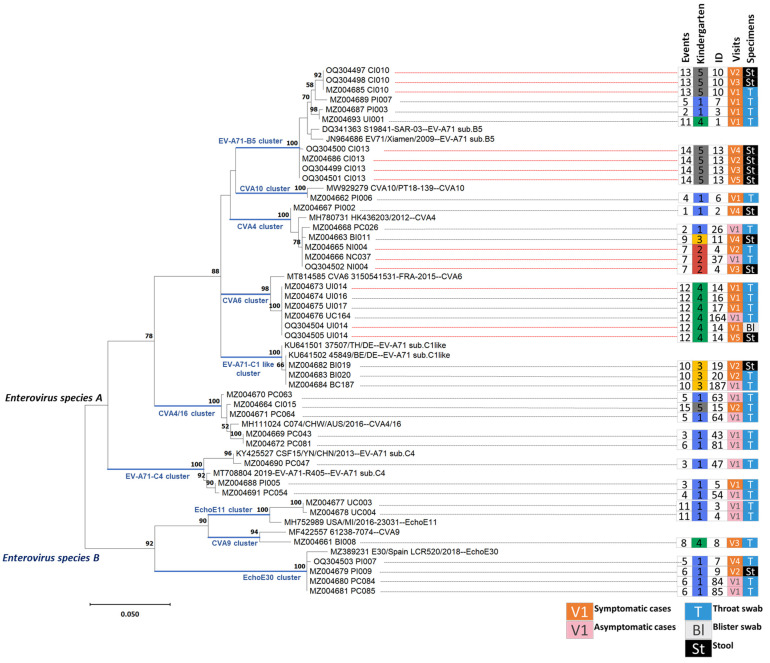
The phylogenetic tree was reconstructed based on 410 bases of the 5′-UTR region from 41 clinical specimens of the selected 18 index cases and 14 case contacts in this cohort study and 13 trimmed complete genome sequences using the maximum likelihood method and General Time-Reversible model. The percentage of trees in which the associated taxa clustered together is shown above the branches. The scale bar indicates 5% mutations per site, equal to a 20.5 single-nucleotide polymorphisms’ distance. The right panel next to the tree shows Event No., followed by Kindergarten No., Identification No., Visiting No., and type of specimens. The boxes showing Visiting No. were filled with orange when specimens belonged to index cases (symptomatic cases) and with pink when specimens belonged to case contacts (asymptomatic cases). The white letter “T” on the blue background represents the throat swab, the black letter “Bl” on the grey background represents the blister swab, and the white letter “St” on the black background represents the stool sample.

Thirteen selected reference sequences representing each enterovirus genotype and specimens from the study cohort were used to reconstruct a tree, as shown in Figure 2. A total of 32 participants (18/22 index cases and 14/25 case contacts) with confirmed enterovirus infections and 41 sequences of high-quality 5′-UTR sequences from all specimens were included in the analysis. Of these thirty-two infections, ten clusters of two species were identified: seven *Enterovirus A* clusters and three *Enterovirus B* clusters (Figure 2). Three of the ten were EV-A71 clusters: the B5, C1-like, and C4 sub-genotype infections. Two further clusters were the CVA4 group separated into CVA4 (closely related to the Hong Kong isolate, MH780731) and CVA4/16 (closely related to the Australian isolate, MH111024). Another two clusters were formed of CVA6 and CVA10. The remaining three clusters were *Enterovirus B* CVA9, EchoE11, and EchoE30.

### 3.2. Statistics of Enterovirus Infections and Manifestations in the Cohort Study

**Figure 3:**
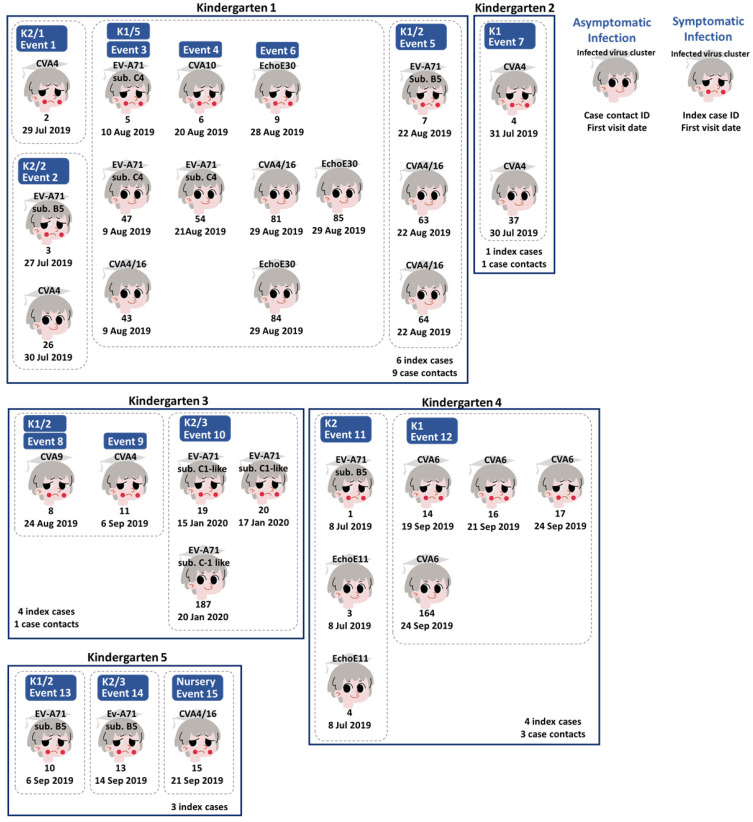
The selected 18 HFMD index cases (symptomatic infections) and 14 case contacts (asymptomatic infections) with confirmed infections via real-time RT-PCR and high-quality 5′-UTR sequences used in this study. A total of 15 infection events were assigned corresponding to index case notifications in each classroom and kindergarten. Each of the five compartments shows the index cases and case contacts in the kindergartens. Dot lines separate participants between classrooms. Each kindergarten’s total number of index cases and case contacts are shown at the bottom of each compartment. Child face characters present infection status. Sad faces with rash (red dots) present symptomatic human enterovirus infections, while happy faces represent asymptomatic infections. The texts above the characters show enterovirus genotypes based on the 5′-UTR sequences. The texts beneath the characters indicate each kindergarten’s participant identification numbers and the date of the first visit.

EV-A71 sub-genotype B5 (two, one, and two cases in Kindergartens 1, 4, and 5, respectively) and CVA4 (two, one, and two cases in Kindergartens 1, 2, and 3, respectively) infections were the most widely observed in the cohort study, followed by CVA4/16 infections (one and two cases in Kindergartens 1 and 5, respectively). The other seven infections were observed only in single kindergartens (Table 1). There were nine, one, one, three, and zero of the fourteen asymptomatic cases identified in Kindergarten 1, 2, 3, 4, and 5, respectively, while six, one, four, four and three of the eighteen infected cases with HFMD or herpangina were identified in Kindergarten 1, 2, 3, 4, and 5, respectively (Figure 3). Cases of HFMD (12/18) were twice as high as the herpangina cases (6/18). EV-A71 sub-genotype B5 and C1-like infections were causes of both HFMD and herpangina (Table 1). CVA4 and EchoE30 were observed in herpangina cases only and not in HFMD, while the other five viruses were observed only in HFMD. HFMD cases were three times higher in males (9/12) than females (3/12), while herpangina cases were found twice as often in females (4/6) than in males (2/6). Most symptomatic cases were mild and self-limiting as they received outpatient care (12/18, 67%) or did not seek hospital care (3/18, 17%). The remaining 3/18 (3%) received inpatient care as the inability to eat caused by pain during chewing or swallowing led to dehydration. There were two cases from Kindergarten 1: an HFMD case infected by CVA10 and a herpangina case infected by EchoE30 (*Enterovirus B*). Additionally, one remaining herpangina case from Kindergarten 2 was infected by CVA4 (Supplementary Data S1).

*Enterovirus A* genotypes were more frequently found than *Enterovirus B* genotypes in symptomatic infections (SI) (16/18–89%) than in asymptomatic infections (ASIs) (10/14--71%). Among ten enterovirus infection clusters in this cohort study, eight were observed as ASIs and SIs (Table 1). The remaining EV-A71 sub-genotype B5 infection caused only SI, and EchoE11 infection caused only ASI. The female proportion of ASIs (9/14, 64%) was higher than for SIs (7/18, 39%). EV-A71 sub-genotype C1-like (one ASI and two SIs), EchoE30 (two ASIs and one SI), and Echo11 (two ASIs) infections were observed only in females, while CVA6 (one ASI, three SIs) and CVA10 (one SI) infections were found only in males. The median ages of all infected cases ranged from three to five years old, except for EV-A71 sub-genotype C1-like infections, where the median age was six years old (Table 1).

### 3.3. Human Enterovirus Infections Circulating in Five Kindergartens

Index cases and case contacts are shown according to geography (kindergartens) and time in Figure 3. An infection event was defined and counted when any symptomatic infections (index cases of HFMD or herpangina) were reported in a given classroom within seven days. From the randomly selected eight classmates of the index cases, participants confirmed as having enterovirus infections via real-time RT PCR (case contacts with asymptomatic infections) were counted in the same event. A total of 15 events were assigned for 12 classrooms of 5 kindergartens (Kindergartens 1 to 5). Of 14 events, a total of 16 symptomatic and 13 asymptomatic participants from 11 classrooms were infected by nine genotypes of *Enterovirus A* and *Enterovirus B* during the endemic wet season (July to September 2019). The remaining events, with two index cases and one asymptomatic case, caused by a single genotype of the EV-A71 sub-genotype C1-like infection, occurred outside the endemic season (January 2020) (Figure 3 and Supplementary Data S1).

In the study, there were six events (6/15, 40%) in which only SIs were identified: Event 1 occurring in Kindergarten 1; Event 8 and Event 9 occurring in the same classroom of Kindergarten 3 14 days apart; and Events 13, 14, and 15 in different classrooms of Kindergarten 5, with Event 14 occurring eight days after Event 13 and Event 15 occurring nine days after Event 14 (Figure 3). Four genotypes caused infections (0.67 per infection): EV-A71 sub-genotype B5 (sub. B5), CVA4, CVA4/16, and CVA9. The CVA9 infection was only found once in the study (Event 8) and closely related to the Taiwan isolate in 2008 (MF422557) with 13 SNP differences (Supplementary Data S3). The CVA4/16 genotype was predominant among ASI in Kindergarten 1, but only one SI of Event 15 in Kindergarten 5 was found. The EV-A71 sub-genotype B5 infections in Events 13 (CI010) and 14 (CI013) occurred in the same kindergarten but were not grouped on the tree with differences of six SNPs (Supplementary Data S4). The CVA4 infections of Events 1 (PI002) and 9 (BI011) occurred in different kindergartens and differed by four SNPs (Figure 3) (Supplementary Data S5).

Two events (2/15, 13%) had more than one index case identified: Event 10 and Event 12 (Figure 3). These two events are examples of classrooms where students were asked to stay home to control the infection transmission according to school policy in Thailand. In Event 10, two index cases and one case contact were infected by the EV-A71 sub-genotype. The C1-like genotype was detected in a single classroom of Kindergarten 3 over two days. The 5′-UTR sequences of three infections (BI019, BI020, and BC187) were identical. It was closely related to reference sequences of German isolates in 2015 (KU641501 and KU641502) and differed from zero to one SNP with two nucleotide insertions. In Event 12, the single genotype of CVA6 caused all infections (three index cases and one case contact were detected in the single classroom of Kindergarten 4 over six days). The 5′-UTR sequences of CVA6 all infections (UI014, UI016, UI017, and UC164) were identical and were closely related to the French isolate collected in 2015 (MT814585), which differed by 6 SNPs. These CVA6 and C1-like sequences were distantly related, with over 20 SNPs difference. The seven events (7/15, 47%) contained one index case and at least one case contact: Events 2, 3, 4, 5, and 6 of Kindergarten 1; Event 7 of Kindergarten 2; and Event 11 of Kindergarten 4 (Figure 3). These were occult infection clusters, since only symptomatic cases were observed.

Events 2, 5, 7, and 11 were single events that occurred in single classrooms. The EV-A71 sub-genotype B5 predominately caused SIs in three events (Events 2, 5, and 11), while ASIs were caused by CVA4 (n = 1), CAV4/16 (n = 2), and EchoE11 (n = 2), respectively. The SIs of EV-A71 sub-genotype B5 in Event 11 (UI001) were clustered together with the SIs in Event 2 (PI003) on the tree with one SNP difference, but differed from the SIs in Event 5 (PI007) by six SNPs (Figure 3) (Supplementary Data S4). Two ASIs of CVA4/16 in Event 5 (PC063 and PC064) were different by two SNPs, while two ASIs of EchoE11 in Event 11 (UC003 and UC004) differed by one SNP. In Event 7 of Kindergarten 2, one SI and one ASI caused two CVA4 with identical 5′-UTR sequences (NI004 and NC037). They were closely related to the SI in Event 9 (BI011) and ASI in Event 2 (PC026) as described above, with differences of one and two SNPs (Figure 3) (Supplementary Data S5).

The series of infections (Events 3, 4, and 6) occurred in a single classroom in Kindergarten 1. In the first event of the series (Event 3), EV-A71 sub-genotype C4 caused only SI and one of two ASIs. In the second event (Event 4), CVA10 caused only SIs, and EV-A71 sub-genotype C4 caused only ASIs. This study’s only detected CVA10 infection was related to a Chinese isolate from 2018 (MW929279) with 4 SNPs difference (Supplementary Data S5). Among EV-A71 sub-genotype C4 infections, the SIs in Event 3 (PI005) were more closely related to the ASIs in Event 4 (PC054) (one SNPs difference) than the ASIs in Event 3 (PC047) (11 SNPs difference). In the last event (Event 6), EchoE30 caused only one SI and two of three ASIs. Additionally, CVA4/16 caused the remaining ASIs in Events 3 and 6. The 5′-UTR sequences of EchoE30 infections were identical and related to the Spanish isolate in 2018 (MZ389231), with five SNPs difference (Supplementary Data S3). The CVA4/16 sequences of the infections in Events 3 and 6 were also identical and related to the Australian isolate in 2016 (MH111024), with five SNPs difference.

### 3.4. Enteroviral RNA Shedding in Throat Swabs and Stool Samples of Index Cases

The proportions of human enterovirus detected in clinical specimens from the index cases during the first visits to the fifth visits (weekly intervals) are shown in Table 2. In total, 4/18 (22%) patients had a negative PCR result during the first visit. However, enterovirus RNAs were identified in throat swabs or stool samples from later visits (Table 2). The remaining 3/18 (17%) patients were uncomfortable giving the throat swab on the first visit. However, they could provide throat swabs or stool samples in the later visits, in which enterovirus RNAs were detected. Of the clinical specimens collected in the first visit, enterovirus was less identified in blister swabs (2/13, 15%) than in throat swabs (11/15, 73%). The trend of enterovirus RNA shedding in the throat and stool samples decreased in the first three and four weeks, respectively. The percentage of the clinical specimens shedding RNA in each weekly interval ranged from 73% to 25% for throat swabs and 71% to 40% for stool samples (Table 2).

Of 18 index cases, there were 11 patients where more than one specimen was positive for the enterovirus real-time RT-PCR assay. Only five had more than one high-quality 5′-UTR sequence, as shown with the grey highlight in Table 2. Almost all 5′-UTR sequences from the same patient were genetically closely related and clustered in the same branch of a given genotype, as shown in Figure 2, even for sequences from different types of specimens (blisters versus throat swabs and throat swabs versus stool samples). For example, the sequences of index case Nos. 4, 14, 10, and 13 were in the CVA4 cluster, CVA6 cluster, and EV-A71 sub. B5 cluster. However, there was only one index case (No. 7) whose throat swab from the 1st and 4th visits contained distantly related RNA of *Enterovirus A* (EV-A71 sub. B5) and *Enterovirus B* (EchoE30), respectively. The 5′-UTR sequence of *Enterovirus B* index case No. 9 and case contact Nos. 84 and 85 from the same kindergarten but in the different classes were closely related, as shown in the EchoE30 cluster (Figure 2) with one SNP difference.

### 3.5. Viral Whole Genome Sequencing for Tracking Echovirus E30 Transmission between Classrooms in the Same Kindergarten

To investigate the transmission of enterovirus using whole genome sequences, throat swabs with virus isolates were selected. Three samples were cultured and used for sequencing with two different methods.

We first compared the whole genome sequences obtained from the same cultured sample using two sequencing protocols: SISPA and direct cDNA sequencing. SISPA sequencing gave a higher mean depth and a higher proportion of the genome covered by 15 reads or more. It was more consistent, with more than 99% of the genome covered by 15 reads or more in all three samples, while this was achieved in only one of three samples via direct cDNA sequencing. A comparison of the base calls between the samples showed 0–2 SNP differences between the sequences generated with different methods. The direct cDNA method’s mean read length was higher than the read using SISPA (Supplementary Figure S1 and Supplementary Data S6).

Whole genome sequences from two samples were analyzed to look for a potential transmission event between these two individuals who had attended different classrooms in Kindergarten 1 (Figure 2). The two samples were the throat swabs taken from Visit 4 of index case No. 7 in class K1/2 and Visit 1 of case contact No. 85 in class K1/5. Analysis of the 5′-UTR sequences in these individuals suggested that both samples were closely related strains of Echo30. The genomes produced via SISPA were used in preference to the direct cDNA method due to the higher coverage of the genome. A pairwise analysis of the two samples showed that there were 46 SNPs which differed between the two samples, which is not suggestive of a direct transmission link between these two individuals.

To rule out the possibility that culture could have introduced mutations, we also performed sequencing on paired isolates from the throat swab taken at Visit 1 of index case No. 3. Samples were prepared using SISPA for both a cultured isolate and directly from the clinical sample. Although the mean depth was low for the clinical sample, and only 52% of the genome was covered to a sufficient depth to call variants confidently, there was only 1 SNP difference between the genome obtained from culture and the genome sequenced directly from the clinical sample.

## 4. Discussion

In this study, we demonstrated that the partial 5′-UTR sequence analysis could infer genotypes and sub-genotypes of human enterovirus in species *Enterovirus A* and *Enterovirus B* directly from clinical specimens. This will be useful for screening enterovirus genotypes quickly after infections are confirmed with an RT-PCR assay. VP1 amplification and sequencing are limited by primer mismatch due to the higher diversity of this protein-coding region compared with the 5′-UTR [36]. Primers specific to enterovirus groups and pre-identification of the enterovirus infection groups are needed to study VP1 diversity and typing independently from virus isolations. Dunn et al. reported the stability of the 5′-UTR sequences from a patient having chronic meningoencephalitis for seven years, showing only a ~0.2% mutation rate per year, suggesting that the 5′-UTR is a stable region of the genome which can be used for genotyping [36].

Most human enterovirus genotypes causing symptomatic infections in this cohort were reported in recent outbreaks in Asia and Southeast Asia. For example, EV-A71, CVA6, and CVA10 are the dominant circulating genotypes in China [37,38]. CVA6-associated HFMD outbreaks have been reported increasingly in Asia and Europe [8,39]. The EV-A71 sub-genotype C4 was a primary target for several licensed vaccines because it was the only dominant genotype identified in the past. Subsequently, new variants of EV-A71, including B5 and C1-like genotypes, emerged and increasingly replaced old variants. This is demonstrated by the proportion of enterovirus reported in northern Vietnam between 2015–2016 [40]: CVA6 (46.7%), EV-A71 B5 (29.9%), and C4 (2.6%) were the dominant genotypes. However, Chu et al. found that the new C4 sub-genotype lineage caused more severe disease than the B5 sub-genotype. The C1-like genotype, emerging from the recombination between the C1 genotype and CVA, has caused several European outbreaks since 2007 as well as outbreaks in Guangdong, China, between 2018 and 2019 [41,42]. In this cohort study, we identified two clusters of CVA4 (CVA4 and CVA4/16) which were closely related to China and Australian sequences in the CVA4 group 2. Besides HFMD and herpangina, CVA4 can cause other clinical manifestations such as acute polyradiculoneuritis, acute flaccid paralysis, severe central nervous system manifestations, or influenza-like illnesses [43,44].

*Enterovirus B* is also associated with severe manifestations such as aseptic meningitis. EchoE30 and EchoE11 caused several aseptic meningitis outbreaks in USA and China [43,45,46,47,48]. EchoE30 caused sporadic to large outbreaks of aseptic meningitis in many regions of the world, such as China [49], Japan [50], and Taiwan [51]. A previous study in Thailand of 1,700 encephalitis cases collected from 2013 to 2018 found that 55 (17.3%) were caused by echoviruses, and EchoE30 was the most common cause of echovirusal encephalitis in the study [52]. Moreover, EchoE30 can be vertically transmitted from mother to child, increasing the difficulty of controlling infections [47,53]. Echoviruses are occasionally detected in HFMD patients [22,39]. In 2005, EchoE11 caused an HFMD outbreak in a Thai hospital nursery (1.1–2.9-year-old average) [54]. However, this cohort study identified CVA9, EchoE11, and EchoE30 in HFMD and herpangina cases or even in children without signs of illness in this cohort study. It suggests that these deadly viruses circulate and are maintained in the environment without necessarily causing an outbreak of severe disease. The surveillance and notification of echovirus disease burden and circulation patterns in Thailand must become active. The high proportion of asymptomatic cases in this study suggests that asymptomatic cases could serve as a reservoir for transmitting infections [55] or for new variants to emerge. These young children with infections could spread the virus to their family members, especially susceptible neonates and young infants who are “children at risk” of severe enterovirus infections [12,56].

Of the infection events, 2/15 (13%) were infections caused by closely genetically related enterovirus clones (no SNP differences found in the 5′-UTR sequences): EV-A71 sub-genotype C1-like in Event 10 and CVA6 in Event 12. These two clones were found once in specific classrooms. Surprisingly, the C1-like clone infected only female children at the highest median age compared with other virus infections in the cohort, while the CVA6 clone caused four cases, all in male children. This might be coincidental, since insufficient data exist to check for a statistically significant result. Usually, small children associate most closely with friends of the same gender in the classroom, which might support disease transmission among interactions between participants in the classrooms. The 6/15 (40%) events which contained only one symptomatic infection in each classroom suggest that good personal or in-classroom hygiene may help to limit transmission in some cases.

The duration of viral shedding is an essential factor in enterovirus disease dynamics. The enterovirus shedding time in the gastrointestinal tract is usually longer than in the respiratory tract [55,57], and more than 20% of cases maintain EV-A71 positivity in stool samples after clearance in their throats [57]. The most prolonged duration of EV-A71 shedding reported in previous studies is 54 days in fecal samples and 30 days in throat swabs, and the duration of EV-A71 shedding is correlated with disease severity [58]. In our study, 66.67%, 71.42%, 50%, and 40% of the stool samples collected at the second, third, fourth, and fifth weeks were real-time RT-PCR-positive for human enterovirus, respectively. Despite the loss of follow-up in throat swab samples, the longest duration of enterovirus shedding in swab samples was four weeks (50%), as described in (Table 2). By weeks 4 and 5, the index case is normally recovered from HFMD symptoms and the child already attending school again. Prolonged virus shedding is a potential risk factor for the HFMD epidemic. A limitation of our study was that we failed to collect throat and stool samples in every follow-up visit; therefore, the data may not be representative enough for the whole cohort. Another limitation is that virus isolation was low in sensitivity and was less successful in stool samples than in throat swabs, which limits our ability to say whether the live virus is shedding from infected patients and asymptomatic infections. Sanitizing in school is an essential factor in HFMD transmission control.

We report the results of whole genome sequencing using Oxford Nanopore Technologies on a small subset of patients in this study. Comparing results obtained from two different methods, we show that both SISPA and direct sequencing of cDNA can be used to obtain complete enterovirus genomes. SISPA gave high coverage and more reliable results, while cDNA gave longer reads and high coverage on one sample but failed to give a complete genome in two samples. Minimal SNP differences were observed between samples generated with different methods, suggesting neither approach gives false positive SNP calls. From this small set of patients, it is impossible to identify factors that predict whether direct cDNA sequencing will be successful, suggesting that SISPA is a more reliable technique when long reads are not absolutely required.

In our study, we saw asymptomatic reinfection of a patient previously infected with a different species strain, closely related to a strain taken from another individual. Using the sequence of the 5′UTR, these two strains had one SNP difference between them. Whole genome sequencing gave a greater resolution of these two strains and allowed us to determine that they differ at 46 positions. This suggests this is not a direct transmission between individuals or kindergarten classrooms but a case of two individuals infected with similar circulating strains. To rule out mutations introduced through the culture process, we compared strains from a patient sample that had been cultured directly from the clinical sample and saw minimal differences between the strains, suggesting that our previous result is not an artefact of the culture process.

Our study reported an asymptomatic reinfection of a patient (index case No. 7) previously infected with a different enterovirus strain. The patient was a three-years-old female who was first infected with EV-A71 sub-genotype B5 and then, after 22 days, reinfected with EchoE30. Due to the lack of cross-protection among different enterovirus subtypes, enterovirus reinfection is quite common and has been reported in many parts of China [59,60,61]. The reinfection episode is separated by an interval of more than 14 days if the previous episode was mild, and more than 23 days if the previous episode was severe [59]. The previously reported population susceptible to HFMD reinfection was males who were younger than four years of age [60]. Moreover, the severity of HFMD was not associated with reinfections or the time interval between HFMD episodes [59]. RNA recombination frequently occurs in enterovirus during co-infection [62,63]. The genetic exchange of enterovirus may lead to the emergence of a new genotype variants, which may lead to significant risks to public health and vaccine development [63]. The co-infection between EV-A71 and CVA16 has been reported previously [35,64]. Our study found that the Sanger sequencing results of the index case No. 7 stool sample from Visit 2 showed double peaks at several positions in the sequence chromatogram which may indicate the presence of co-infection between EV-A71 and EchoE30.

Overall, these results demonstrate the diversity and complexity of human enterovirus circulating within and between classrooms/kindergartens during the endemic season, in which only a few groups cause disease. We demonstrate that the 5′-UTR is an alternative target for genotyping, with advantages over the traditional VP1 region, as the 5′-UTR has sufficient diversity to be used for typing without excess diversity, which hampers attempts to design pan-genotype primers in VP1. While we were able to genotype strains based on partial Sanger sequences, we also demonstrate that whole genome sequencing is a valuable tool for determining transmission at higher resolutions than those provided by partial sequences and ruling out a possible transmission event between individuals. Together, these results show that deeper investigation into the dynamics of enterovirus circulation, combined with genomic sequencing strategies to determine strains and genotypes, would be beneficial to target interventions to reduce disease transmission from kindergartens to susceptible family members (or vice versa) through young children. Active surveillance of genotypes of enterovirus circulating in pregnant women and young children at the national level during epidemic seasons could be placed into the strategic plan to control severe diseases caused by severe emerging enterovirus infections.

## Figures and Tables

**Table 1:** Gender ratio, median age, and distribution of cases in each of the five kindergartens in the Bangkok metropolitan area, Thailand, between June 2019 to January 2020 stratified by enterovirus genotypes causing asymptomatic and symptomatic infections, including HFMD or herpangina.

Enterovirus Clusters	^a^ Infection Distributions (K. 1-2-3-4-5)	^b^ Age (Min–Max)	^c^ Participants with ASI (Ratio F:M)	Symptomatic Infections		
				^d^ All Participants (Ratio F:M)	^e^ HFMD (Ratio F:M)	^f^ Herpangina (Ratio F:M)
**Total Number**	**5 (15-2-5-7-3)**		**14 (9:5)**	**18 (7:11)**	**12 (3:9)**	**6 (4:2)**
***Enterovirus A***						
EV-A71 sub. B5	3 (2-0-0-1-2)	4 (3–5)	N/A	5 (1:4)	4 (1:3)	1 (0:1)
EV-A71 sub. C1-like	1 (0-0-3-0-0)	6 (5–6)	1 (1:0)	2 (2:0)	1 (1:0)	1 (1:0)
EV-A71 sub. C4	1 (3-0-0-0-0)	4 (3-5)	2 (1:1)	1 (0:1)	1 (0:1)	N/A
CVA4	3 (2-2-1-0-0)	4 (3–5)	2 (1:1)	3 (2:1)	N/A	3 (2:1)
CVA4/16	2 (4-0-0-0-1)	3 (3–4)	4 (2:2)	1 (0:1)	1 (0:1)	N/A
CVA6	1 (0-0-0-4-0)	4 (4–4)	1 (0:1)	3 (0:3)	3 (0:3)	N/A
CVA10	1 (1-0-0-0-0)	3 (N/A)	N/A	1 (0:1)	1 (0:1)	N/A
***Enterovirus B***						
CVA9	1 (0-0-1-0-0)	4 (N/A)	N/A	1 (1:0)	1 (1:0)	N/A
EchoE30	1 (3-0-0-0-0)	3.5 (3–4)	2 (2:0)	1 (1:0)	N/A	1 (1:0)
EchoE11	1 (0-0-0-2-0)	5 (5–5)	2 (2:0)	N/A	N/A	N/A

^a^ Numbers indicate the total number of kindergartens where the virus infections were observed. The five digits in the parentheses are the number of given virus infections found in each of the five kindergartens in order from Kindergarten 1 to 5, separated with a dash. ^b^ Median age (in years) of participants infected with each virus group. The minimum and maximum age in each group is shown in the paratheses. The number indicates the total number of participants with asymptomatic infections (ASIs) ^c^, symptomatic infections ^d^, hand–foot–mouth disease (HFMD) ^e^, and herpangina ^f^. The ratio of female (F)/male (M) cases is shown in the paratheses.

**Table 2:** The presence and genotype of enterovirus in clinical specimens from each of the 18 index cases of the 15 events. During the 1st visit, blister and throat swabs were collected, while throat swabs and stool samples were collected four times weekly after the 1st visit. Enterovirus genotypes inferred from specimens with the real-time RT-PCR positive results and high-quality results of Sanger sequencing in both directions (grey highlight).

Event No.	Index Case No.	Enterovirus Genotypes	Blister Swabs ^c^	Quantitative Cycles of RT-PCR of Throat Swabs at Visit					Quantitative Cycles of RT-PCR of Stool Samples at Visit			
				1	2	3	4	5	2	3	4	5
1	2	CVA4	-	Neg	-	Neg	Neg	Neg	-	-	22.53	Neg
2	3	EV-A71 sub. B5	-	19.91 ^a,d^	-	32.97	35.20	Neg	-	-	32.01	Neg
3	5	EV-A71 sub. C4	Neg	25.08 ^a^	-	-	-	-	-	-	-	23.54
4	6	CVA10	Neg	25.50	-	-	-	Neg	-	-	Neg	-
5	7	EV-A71 sub. B5 and EchoE30 ^b^	Neg	28.70 ^a,d^	-	Neg	29.73 ^a,d^	-	24.48	-	Neg	Neg
6	9	EchoE30	-	-	-	-	-	-	22.04	34.77	-	Neg
7	4	CVA4	-	28.16	29.40	Neg	34.50	-	-	35.73	38.49	35.57
8	8	CVA9	Neg	Neg	Neg	24.79	-	-	-	-	-	-
9	11 ^a^	CVA4	Neg	Neg	-	-	-	-	Neg	Neg	32.15	-
10	19	EV-A71 sub. C1-like	26.02	-	Neg	Neg	Neg	-	36.21	-	-	-
10	20 ^a^	EV-A71 sub. C1-like	-	-	23.89	Neg	Neg	-	-	23.36	31.17	-
11	1	EV-A71 sub. B5	Neg	29.70	-	Neg	-	Neg	-	-	-	-
12	14	CVA6	35.38 & 20.75 ^c^	29.11	-	-	-	-	Neg	-	Neg	23.02
12	16	CVA6	Neg	30.48	-	-	-	-	33.41	-	-	-
12	17	CVA6	Neg	32.04	-	-	-	-	-	-	-	-
13	10	EV-A71 sub. B5	Neg	34.71	-	-	-	-	22.58	32.55	Neg	Neg
14	13	EV-A71 sub. B5	Neg	Neg	-	-	-	-	25.06	28.35	29.07	30.98
15	15	CVA4/16	Neg	34.58	-	-	-	-	Neg	Neg	Neg	Neg
**A total of the virus-detected specimens** **(%)**			2/13 (15)	11/15 (73)	2/4 (50)	2/8 (25)	3/6 (50)	0/4 (0)	6/9 (67)	5/7 (71)	6/11 (55)	4/10 (40)

^a^ All specimens were taken for virus isolation. These specimens were culture-positive. ^b^ This case was infected by two different genotypes and species: symptomatic infection by EV-A71 sub. B5 of *Enterovirus A* found in throat swabs from Visit 1 and asymptomatic infection by EchoE30 of *Enterovirus B* found in throat swabs from Visit 4. ^c^ Two blister swabs were collected from participants each time. This participant showed two real-time RT-PCR positives for both specimens with intermediate (20 < Cq < 30) and low (Cq < 30) RNA virus loads. ^d^ These cultures and specimens were undertaken for genome sequencing.

## Data Availability

Viral 5′-UTR sequences were submitted to GenBank; see details in Supplementary Data S1. The genome virus was submitted to the United States National Center for Biotechnology Information (NCBI) as BioProject ID PRJNA974406.

## Supplementary Materials

The following supporting information can be downloaded at: https://www.mdpi.com/article/10.3390/v15061397/s1. Supplementary Data S1: Participants’ demographic data and specimens associated with laboratory results. Supplementary Data S2: Metadata of this cohort’s partial 5′-UTR sequences and complete genome sequences acquired from the GenBank database. Supplementary Data S3: 5′-UTR SNPs difference among *Enterovirus B*. Supplementary Data S4: 5′-UTR SNPs difference among EV-A71. Supplementary Data S5: 5′-UTR SNPs difference among Coxsackieviruses. Supplementary Data S6: Statistics of the whole genome sequencing read comparison between the SISPA-based method and cDNA direct sequencing using Oxford Nanopore Technology. Supplementary Figure S1: Graphs showing genome coverages, mean sequencing depth, and mean read length in comparison of index case No. 7 and case contact No. 85 infected with EchoE30.

## References

[1] Corsino C.B., Ali R., Linklater D.R. (2023). Herpangina. StatPearls.

[2] Li W., Gao H.-H., Zhang Q., Liu Y.-J., Tao R., Cheng Y.-P., Shu Q., Shang S.-Q. (2016). Large outbreak of herpangina in children caused by enterovirus in summer of 2015 in Hangzhou, China. Sci. Rep..

[3] de Ceano-Vivas M., Garcia M.L., Velazquez A., Martin Del Valle F., Menasalvas A., Cilla A., Epalza C., Romero M.P., Cabrerizo M., Calvo C. (2021). Neurodevelopmental Outcomes of Infants Younger Than 90 Days Old Following Enterovirus and Parechovirus Infections of the Central Nervous System. Front. Pediatr..

[4] Olchawa-Czech A., Ptak K., Szymonska I., Kwinta P. (2021). Severe enterovirus infections in infants <3 months of age and the importance of medical history. J. Mother. Child..

[5] Yang X., Duan L., Zhan W., Tang Y., Liang L., Xie J., Luo M. (2023). Enterovirus B types cause severe infection in infants aged 0–3 months. Virol. J..

[6] Weng Y., Chen W., He W., Huang M., Zhu Y., Yan Y. (2017). Serotyping and Genetic Characterization of Hand, Foot, and Mouth Disease (HFMD)-Associated Enteroviruses of No-EV71 and Non-CVA16 Circulating in Fujian, China, 2011–2015. Med. Sci. Monit. Int. Med. J. Exp. Clin. Res..

[7] Nhan L.N.T., Turner H.C., Khanh T.H., Hung N.T., Lien L.B., Hong N.T.T., Nhu L.N.T., Ny N.T.H., Nguyet L.A., Thanh T.T. (2019). Economic Burden Attributed to Children Presenting to Hospitals with Hand, Foot, and Mouth Disease in Vietnam. Open Forum Infect. Dis..

[8] Lerdsamran H., Prasertsopon J., Mungaomklang A., Klinmalai C., Noisumdaeng P., Sangsiriwut K., Tassaneetrithep B., Guntapong R., Iamsirithaworn S., Puthavathana P. (2018). Seroprevalence of antibodies to enterovirus 71 and coxsackievirus A16 among people of various age groups in a northeast province of Thailand. Virol. J..

[9] Linsuwanon P., Puenpa J., Huang S.-W., Wang Y.-F., Mauleekoonphairoj J., Wang J.-R., Poovorawan Y. (2014). Epidemiology and seroepidemiology of human enterovirus 71 among Thai populations. J. Biomed. Sci..

[10] Puenpa J., Auphimai C., Korkong S., Vongpunsawad S., Poovorawan Y. (2018). Enterovirus A71 Infection, Thailand, 2017. Emerg. Infect. Dis..

[11] Puenpa J., Chieochansin T., Linsuwanon P., Korkong S., Thongkomplew S., Vichaiwattana P., Theamboonlers A., Poovorawan Y. (2013). Hand, foot, and mouth disease caused by coxsackievirus A6, Thailand, 2012. Emerg. Infect. Dis..

[12] Organisation W.H. Enterovirus Infection—France. https://www.who.int/emergencies/disease-outbreak-news/item/2023-DON469.

[13] Chen M., Zuo X., Tan Y., Ju Y., Bi F., Wang H., Chen M. (2019). Six amino acids of VP1 switch along with pandemic of CV-A6-associated HFMD in Guangxi, southern China, 2010–2017. J. Infect..

[14] Song Y., Zhang Y., Ji T., Gu X., Yang Q., Zhu S., Xu W., Xu Y., Shi Y., Huang X. (2017). Persistent circulation of Coxsackievirus A6 of genotype D3 in mainland of China between 2008 and 2015. Sci. Rep..

[15] Yang X., Li Y., Zhang C., Zhan W., Xie J., Hu S., Chai H., Liu P., Zhao H., Tang B. (2020). Clinical features and phylogenetic analysis of severe hand-foot-and-mouth disease caused by Coxsackievirus A6. Infect. Genet. Evol..

[16] Gauthier N.P.G., Nelson C., Bonsall M.B., Locher K., Charles M., MacDonald C., Krajden M., Chorlton S.D., Manges A.R. (2021). Nanopore metagenomic sequencing for detection and characterization of SARS-CoV-2 in clinical samples. PLoS ONE.

[17] Lewandowski K., Xu Y., Pullan S.T., Lumley S.F., Foster D., Sanderson N., Vaughan A., Morgan M., Bright N., Kavanagh J. (2019). Metagenomic Nanopore Sequencing of Influenza Virus Direct from Clinical Respiratory Samples. J. Clin. Microbiol..

[18] Wollants E., Maes P., Merino M., Bloemen M., Van Ranst M., Vanmechelen B. (2020). First genomic characterization of a Belgian Enterovirus C104 using sequence-independent Nanopore sequencing. Infect. Genet. Evol..

[19] Zakotnik S., Korva M., Knap N., Robnik B., Gorišek Miksić N., Avšič Županc T. (2019). Complete Coding Sequence of a Chikungunya Virus Strain Imported into Slovenia from Thailand in Late 2018. Microbiol. Resour. Announc..

[20] Zhang L., Zhao L., Zhang Z., Hong W., Wang J., Qiu S., Yang H., Gan M., Sun J., Zhao J. (2021). Genetic and pathogenicity diversity of dengue virus type 2 strains circulating in Guangdong, China. Biosaf. Health.

[21] Delahaye C., Nicolas J. (2021). Sequencing DNA with nanopores: Troubles and biases. PLoS ONE.

[22] Thammasonthijarern N., Kosoltanapiwat N., Nuprasert W., Sittikul P., Sriburin P., Pan-Ngum W., Maneekan P., Hataiyusuk S., Hattasingh W., Thaipadungpanit J. (2021). Molecular Epidemiological Study of Hand, Foot, and Mouth Disease in a Kindergarten-Based Setting in Bangkok, Thailand. Pathogens.

[23] Lekana-Douki S.E., Sir-Ondo-Enguier P.N., Banga-Mve-Ella O., Imboumy-Limoukou R.K., Maganga G.D., Lekana-Douki J.B., Berthet N. (2019). Epidemiology and molecular characterization of the re-emerging measles virus among children and adults in the Haut-Ogooue, Gabon. BMC Infect. Dis..

[24] Kumar S., Stecher G., Li M., Knyaz C., Tamura K. (2018). MEGA X: Molecular Evolutionary Genetics Analysis across Computing Platforms. Mol. Biol. Evol..

[25] Greninger A.L., Naccache S.N., Federman S., Yu G., Mbala P., Bres V., Stryke D., Bouquet J., Somasekar S., Linnen J.M. (2015). Rapid metagenomic identification of viral pathogens in clinical samples by real-time nanopore sequencing analysis. Genome Med..

[26] Grüning B., Dale R., Sjödin A., Chapman B.A., Rowe J., Tomkins-Tinch C.H., Valieris R., Köster J. (2018). Bioconda: Sustainable and comprehensive software distribution for the life sciences. Nat. Methods.

[27] Martin M. (2011). Cutadapt removes adapter sequences from high-throughput sequencing reads. EMBnet. J..

[28] De Coster W., D’Hert S., Schultz D.T., Cruts M., Van Broeckhoven C. (2018). NanoPack: Visualizing and processing long-read sequencing data. Bioinformatics.

[29] Li H. (2018). Minimap2: Pairwise alignment for nucleotide sequences. Bioinformatics.

[30] Li H. (2021). New strategies to improve minimap2 alignment accuracy. Bioinformatics.

[31] Li H., Handsaker B., Wysoker A., Fennell T., Ruan J., Homer N., Marth G., Abecasis G., Durbin R. (2009). The Sequence Alignment/Map format and SAMtools. Bioinformatics.

[32] Danecek P., Bonfield J.K., Liddle J., Marshall J., Ohan V., Pollard M.O., Whitwham A., Keane T., McCarthy S.A., Davies R.M. (2021). Twelve years of SAMtools and BCFtools. Gigascience.

[33] Quinlan A.R., Hall I.M. (2010). BEDTools: A flexible suite of utilities for comparing genomic features. Bioinformatics.

[34] Shen W., Le S., Li Y., Hu F. (2016). SeqKit: A Cross-Platform and Ultrafast Toolkit for FASTA/Q File Manipulation. PLoS ONE.

[35] Liu M.Y., Liu J., Lai W., Luo J., Liu Y., Vu G.P., Yang Z., Trang P., Li H., Wu J. (2016). Characterization of enterovirus 71 infection and associated outbreak of Hand, Foot, and Mouth Disease in Shawo of China in 2012. Sci. Rep..

[36] Dunn J.J., Romero J.R., Wasserman R., Rotbart H.A. (2000). Stable Enterovirus 5′ Nontranslated Region over a 7-Year Period in a Patient with Agammaglobulinemia and Chronic Infection. J. Infect. Dis..

[37] Chen M., He S., Yan Q., Xu X., Wu W., Ge S., Zhang S., Chen M., Xia N. (2017). Severe hand, foot and mouth disease associated with Coxsackievirus A10 infections in Xiamen, China in 2015. J. Clin. Virol..

[38] Yang F., Yuan J., Wang X., Li J., Du J., Su H., Zhou B., Jin Q. (2014). Severe Hand, Foot, and Mouth Disease and Coxsackievirus A6—Shenzhen, China. Clin. Infect. Dis..

[39] Noisumdaeng P., Korkusol A., Prasertsopon J., Sangsiriwut K., Chokephaibulkit K., Mungaomklang A., Thitithanyanont A., Buathong R., Guntapong R., Puthavathana P. (2019). Longitudinal study on enterovirus A71 and coxsackievirus A16 genotype/subgenotype replacements in hand, foot and mouth disease patients in Thailand, 2000–2017. Int. J. Infect. Dis..

[40] Chu S.T., Kobayashi K., Bi X., Ishizaki A., Tran T.T., Phung T.T.B., Pham C.T.T., Nguyen L.V., Ta T.A., Khu D.T.K. (2020). Newly emerged enterovirus-A71 C4 sublineage may be more virulent than B5 in the 2015–2016 hand-foot-and-mouth disease outbreak in northern Vietnam. Sci. Rep..

[41] Liu Y., Zhou J., Ji G., Gao Y., Zhang C., Zhang T., Huo J., Liang W., Yang J., Shi Y. (2022). A novel subgenotype C6 Enterovirus A71 originating from the recombination between subgenotypes C4 and C2 strains in mainland China. Sci. Rep..

[42] Zeng H., Yi L., Chen X., Zhou H., Zheng H., Lu J., Yang F., Li C., Fang L., Zhang X. (2021). Emergence of a non vaccine-cognate enterovirus A71 genotype C1 in mainland China. J. Infect..

[43] Li J., Ni N., Cui Y., Zong S., Yao X., Hu T., Cao M., Zhang Y., Hou P., Carr M.J. (2022). An outbreak of a novel recombinant Coxsackievirus A4 in a kindergarten, Shandong province, China, 2021. Emerg. Microbes Infect..

[44] Wang M., Li J., Yao M.-X., Zhang Y.-W., Hu T., Carr M.J., Duchêne S., Zhang X.-C., Zhang Z.-J., Zhou H. (2019). Genome Analysis of Coxsackievirus A4 Isolates from Hand, Foot, and Mouth Disease Cases in Shandong, China. Front. Microbiol..

[45] Brouwer L., Moreni G., Wolthers K.C., Pajkrt D. (2021). World-Wide Prevalence and Genotype Distribution of Enteroviruses. Viruses.

[46] CDC Outbreaks of Aseptic Meningitis Associated with Echoviruses 9 and 30 and Preliminary Surveillance Reports on Enterovirus Activity—United States, 2003. https://www.cdc.gov/mmwr/preview/mmwrhtml/mm5232a1.htm.

[47] Li J., Yan D., Chen L., Zhang Y., Song Y., Zhu S., Ji T., Zhou W., Gan F., Wang X. (2019). Multiple genotypes of Echovirus 11 circulated in mainland China between 1994 and 2017. Sci. Rep..

[48] Tian X., Han Z., He Y., Sun Q., Wang W., Xu W., Li H., Zhang Y. (2021). Temporal phylogeny and molecular characterization of echovirus 30 associated with aseptic meningitis outbreaks in China. Virol. J..

[49] Xiao H., Guan D., Chen R., Chen P., Monagin C., Li W., Su J., Ma C., Zhang W., Ke C. (2013). Molecular characterization of echovirus 30-associated outbreak of aseptic meningitis in Guangdong in 2012. Virol. J..

[50] Akiyoshi K., Nakagawa N., Suga T. (2007). An outbreak of aseptic meningitis in a nursery school caused by echovirus type 30 in Kobe, Japan. Jpn. J. Infect. Dis..

[51] Zheng S., Ye H., Yan J., Xie G., Cui D., Yu F., Wang Y., Yang X., Zhou F., Zhang Y. (2016). Laboratory diagnosis and genetic analysis of a family clustering outbreak of aseptic meningitis due to echovirus 30. Pathog. Glob. Health.

[52] Hemachudha P., Petcharat S., Hinjoy S., Saraya A.W., Hemachudha T. (2021). Encephalitis in Thailand: A Neglected Disease Increasingly Caused by Enterovirus. Trop. Med. Infect. Dis..

[53] Nagington J., Wreghittt T.G., Gandy G., Roberton N.R., Berry P.J. (1978). Fatal echovirus 11 infections in outbreak in special-care baby unit. Lancet.

[54] Apisarnthanarak A., Kitphati R., Pongsuwann Y., Tacharoenmueng R., Mundy L.M. (2005). Echovirus type 11: Outbreak of hand-foot-and-mouth disease in a Thai hospital nursery. Clin. Infect. Dis..

[55] Lim C.T., Jiang L., Ma S., James L., Ang L.W. (2016). Basic reproduction number of coxsackievirus type A6 and A16 and enterovirus 71: Estimates from outbreaks of hand, foot and mouth disease in Singapore, a tropical city-state. Epidemiol. Infect..

[56] Marotta C., Di Gennaro F., Pizzol D., Madeira G., Monno L., Saracino A., Putoto G., Casuccio A., Mazzucco W. (2018). The At Risk Child Clinic (ARCC): 3 Years of Health Activities in Support of the Most Vulnerable Children in Beira, Mozambique. Int. J. Environ. Res. Public Health.

[57] Han J., Ma X.-J., Wan J.-F., Liu Y.-H., Han Y.-L., Chen C., Tian C., Gao C., Wang M., Dong X.-P. (2010). Long persistence of EV71 specific nucleotides in respiratory and feces samples of the patients with Hand-Foot-Mouth Disease after recovery. BMC Infect. Dis..

[58] Li J., Lin C., Qu M., Li X., Gao Z., Zhang X., Liu Y., Huang Y., Wang X., Jia L. (2013). Excretion of enterovirus 71 in persons infected with hand, foot and mouth disease. Virol. J..

[59] Huang J., Liao Q., Ooi M.H., Cowling B.J., Chang Z., Wu P., Liu F., Li Y., Luo L., Yu S. (2018). Epidemiology of Recurrent Hand, Foot and Mouth Disease, China, 2008–2015. Emerg. Infect. Dis..

[60] Shi C., Liu J., Shi P., Ji H., Shen Y., Qian Y.H. (2018). Epidemiological characteristics and influential factors of hand, foot, and mouth disease reinfection in Wuxi, China, 2008–2016. BMC Infect. Dis..

[61] Zhong X., Wang H., Chen C., Zou X., Li T. (2022). Epidemiological Characteristics of Hand, Foot and Mouth Disease Reinfection in Guangzhou, Southern China from 2012 to 2017. Iran J. Public Health.

[62] Muslin C., Mac Kain A., Bessaud M., Blondel B., Delpeyroux F. (2019). Recombination in Enteroviruses, a Multi-Step Modular Evolutionary Process. Viruses.

[63] Zhao H., Wang J., Chen J., Huang R., Zhang Y., Xiao J., Song Y., Ji T., Yang Q., Zhu S. (2022). Molecular Epidemiology and Evolution of Coxsackievirus A9. Viruses.

[64] Liu M.Y., Liu W., Luo J., Liu Y., Zhu Y., Berman H., Wu J. (2011). Characterization of an outbreak of hand, foot, and mouth disease in Nanchang, China in 2010. PLoS ONE.

