# Supplementary figures and images for: Diversity of Human Enterovirus Co-Circulations in Five Kindergartens in Bangkok between July 2019 and January 2020
Source: Viruses. 2023 Jun 20;15(6):1397. doi: 10.3390/v15061397 (PMC10301176; doi:10.3390/v15061397)

A

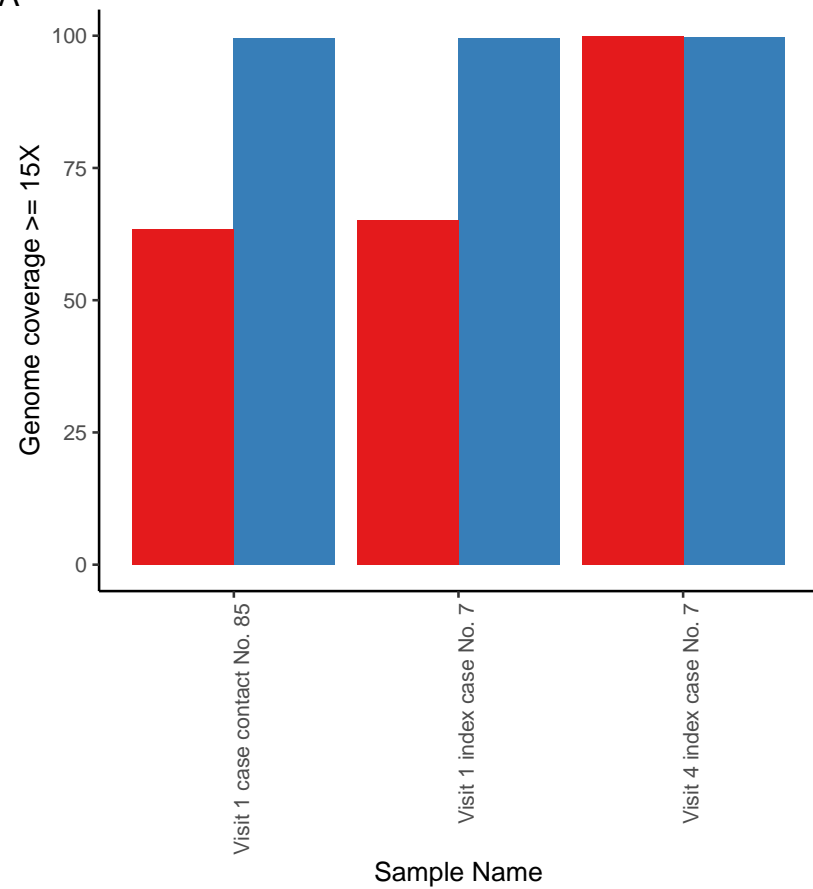

B

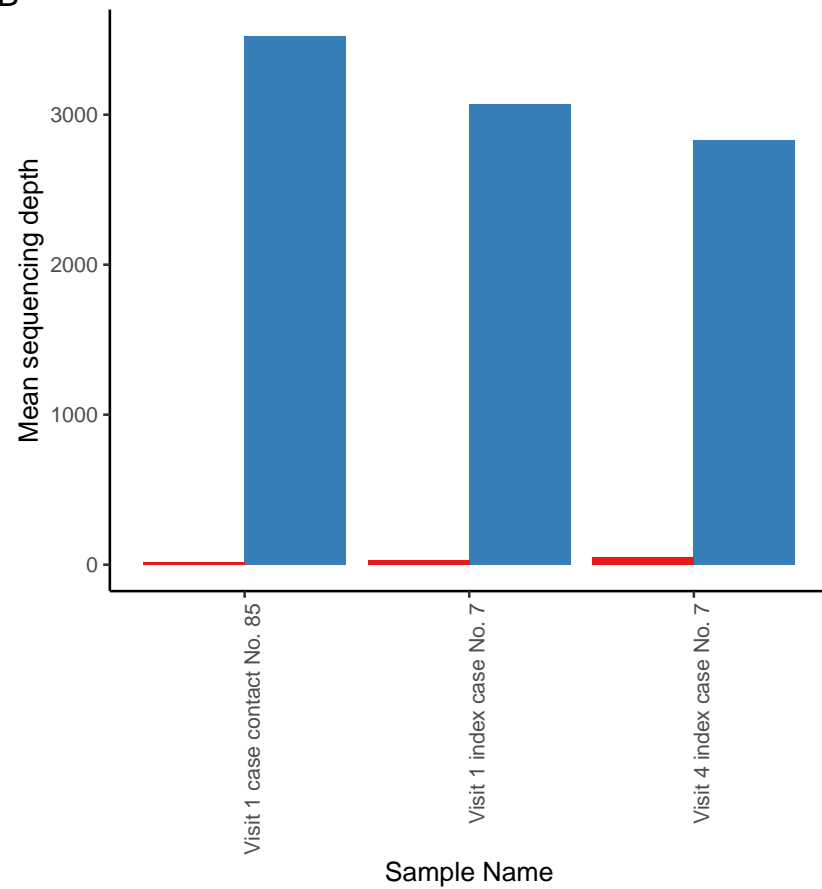

C

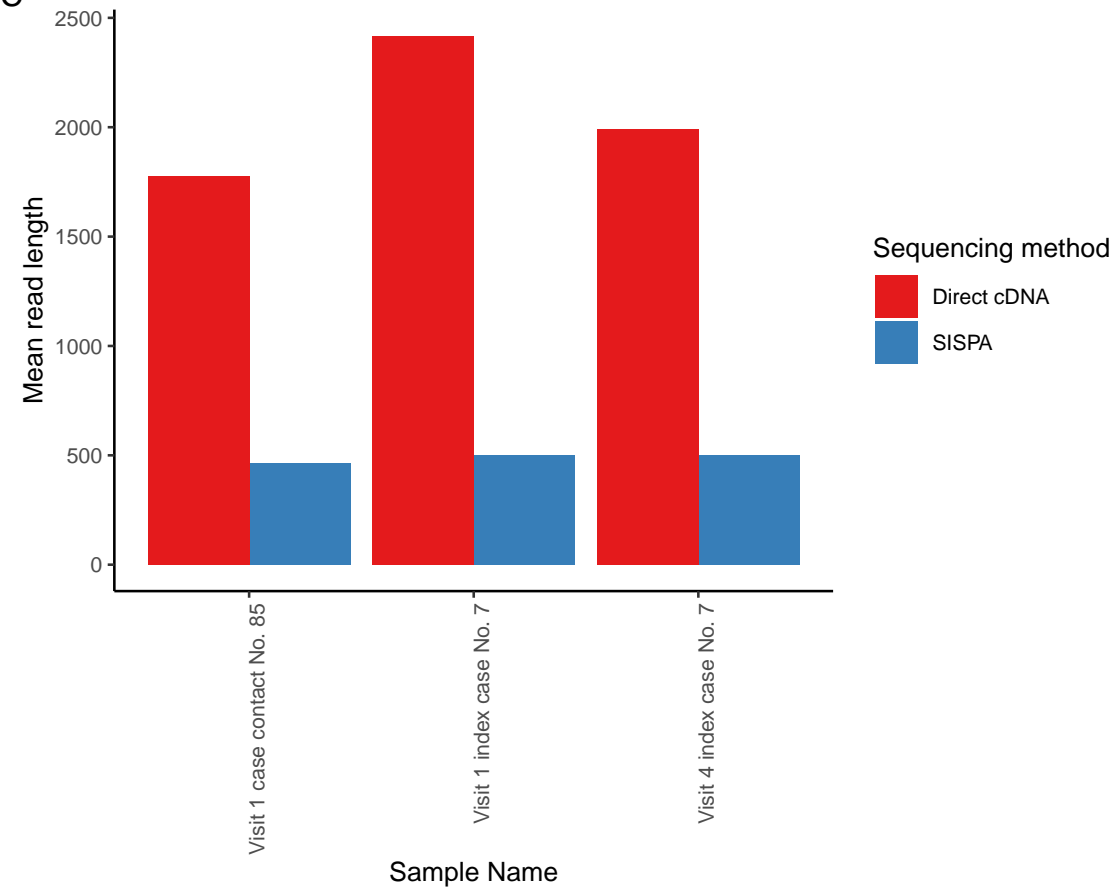

Supplement: Supplementary file 1 [file viruses-15-01397-s001.zip › Supplementary_Figure_S1_WGS_No7n85.pdf]
